# The Predictive Role of Inflammatory Biomarkers in Atrial Fibrillation as Seen through Neutrophil-Lymphocyte Ratio Mirror

**DOI:** 10.1155/2016/8160393

**Published:** 2016-07-03

**Authors:** Feliciano Chanana Paquissi

**Affiliations:** Department of Medicine, Clínica Girassol, Luanda, Angola

## Abstract

Atrial fibrillation (AF) is the most common arrhythmia and is responsible for significant disease burden worldwide. Current evidence has suggested that systemic inflammatory response plays a crucial role in the initiation, maintenance, and progression of AF. So, recent efforts have been directed in search of measurable inflammatory biomarkers as additional tools in severity and prognosis assessment of AF. A simple, and easily obtainable, inflammatory marker is the neutrophil-lymphocyte ratio (NLR), which has shown good performance in preliminary studies as a potential prognostic biomarker in patients with AF. In this work, we performed a thorough review of clinical studies that evaluated the role of C-reactive protein (CRP), interleukin-6 (IL-6), and NLR as predictors of outcomes in AF. We gave a particular emphasis on the NLR because it is a simpler, widely available, and inexpensive biomarker.

## 1. Introduction

Atrial fibrillation (AF) is the most common cardiac arrhythmia, which in 2010 was estimated to affect about 33.5 million individuals in the world [1], with a prevalence around 2.3–3.4% among adults [2], and may reach 9% in those aged over 80 years [3]. Studies point to an increase in both incidence and prevalence [1, 4, 5], as well as the attributable mortality [1, 5], so that between 1990 and 2013 AF was the factor with the greatest relative increase in the burden of cardiovascular diseases (CVDs) [5]. Future projections predict a 2-fold increase in the number of cases of AF in 2050 [3]. The AF is associated with significant morbidity and mortality, increasing the risk of stroke and death from all causes [6, 7].

Recent investigations have registered significant advances in the understanding of the pathogenic mechanisms underlying AF [8, 9]. One of the most explored mechanisms and that has gained more and more space in recent years is the inflammatory response [10, 11]. The literature has emphasized the role of inflammation in the initiation, maintenance, and progression of AF [8, 12, 13]. Several inflammatory biomarkers, as C-reactive protein (CRP) and interleukins, have been associated with the occurrence of AF and its prognosis, including vascular events [10, 14–16]. In fact, the incidence of AF is increased in other situations that share significant systemic inflammatory response such as nonalcoholic fatty liver disease [17] and metabolic syndrome [18, 19], suggesting a role of inflammation as a mediator between atrial fibrillation and these situations.

Among the inflammatory biomarkers, the neutrophil-lymphocyte ratio (NLR), defined as the ratio of absolute counts of neutrophils and lymphocytes, has emerged recently as effective outcomes predictor in atrial fibrillation [20, 21], a role also demonstrated for ischemic heart disease and stroke [22–24]. NLR is a simple, inexpensive, and widely available biomarker and has been shown to be a good predictor of atrial arrhythmias that reflects the role of unbalanced white cells (with the predominance of activated neutrophils) in arrhythmogenesis [8, 9, 25].

Multiple inflammatory markers have been studied as predictors of outcomes in AF, from those with potential direct involvement in the pathogenesis, such as IL-6 and NLR [11, 26], and others only as a reflection of underlying immune responses, but apparently without direct participation as CRP [27]. There are those that are being more used in research contexts than in clinical practice. In this paper, we performed a thorough review of clinical studies focusing on CRP, IL-6, and NLR, as they are more reliable in clinical practice, with particular emphasis on the NLR because it is simpler, more widely available, and inexpensive.

## 2. Overview of Inflammatory Biomarkers in Atrial Fibrillation

Several studies have demonstrated the association between inflammatory markers and the incidence, severity, response to treatment, and prognosis in AF [14–16, 28]. An analysis of a large study with 17,120 participants, without prior history of arrhythmia, high-sensitivity C-reactive protein (hs-CRP), was associated with a 36% increase in the risk of developing AF (hazard ratio [HR]: 1.37, *p*-trend < 0.01) for each increasing tertile above baseline, with persistently high risk, comparing the highest to the lowest hs-CRP tertile, even after adjustment for potential confounders (hazard ratio [HR] 1.96; *p* < 0.01) [29]. In other studies in patients with AF, CRP was a significant predictor of stroke [14, 30] and peripheral embolism [31]. In treated patients who underwent electrical cardioversion, a high hs-CRP was an independent predictor of AF recurrence even after adjusting for confounders variables [32, 33]. In another study following patients after catheter ablation, a high hs-CRP was an independent predictor of recurrence (*p* = 0.021) during a median follow-up of 15 months [15]. The contribution of inflammation in atrial activity seems to begin early, as demonstrated in a study where a high CRP was an independent risk factor for spontaneous contrast in transesophageal echocardiography [34]. This reflects that electromechanical impairment begins before any electrocardiographic visible dysrhythmia.

Other inflammatories biomarkers associated with AF and its progression are interleukin-6 (IL-6) [35] and interleukin-18 [36]. In one of these studies, with 3,762 adults with chronic kidney disease, a high plasma IL-6 level was associated with AF at baseline (Odds Ratio [OR], 1.61; *p* = 0.001) and predicted new-onset AF (OR, 1.25; *p* = 0.03) during a mean follow-up of 3.7 years [35]. In a study with patient in oral anticoagulation for AF, a high-sensitivity interleukin-6 (hsIL6) was a predictor of long-term cardiovascular events (HR 1.97, *p* = 0.002) and all-cause mortality (HR 2.48, *p* < 0.001) [37]; and adding hsIL6 to the clinical risk scores (CHADS2 and CHA2DS2-VASc) improved the discrimination index value for prediction of long-term cardiovascular events and death [37]. In another study with rhythm control strategy, IL-6 and CRP were significantly higher in those with AF recurrence than in those maintaining sinus rhythm (mean IL-6: 1.84 versus 1.19, *p* < 0.005; CRP: 1.24 versus 0.59, *p* < 0.005) [38].  Table 1 summarizes the clinical studies that have assessed the role of general inflammatory biomarkers in AF.

## 3. The Particular Role of Neutrophil-Lymphocyte Ratio as a Predictive Biomarker in Atrial Fibrillation

### 3.1. Incidence and Prevalence

A high NLR is associated with increased incidence of AF, as was evident in a prospective cohort, with 275 patients who underwent nonemergency coronary artery bypass grafting, where the group with postoperative AF had higher preoperative NLR (median 3.0 versus 2.4, *p* = 0.001) [21]. These findings were also evident in a study with patients undergoing coronary angiography, with stent placement, for acute ST-segment elevation myocardial infarction, where those who developed AF had higher postcatheterization NLRs at 48 hours (median 5.23 versus 3.00, *p* = 0.05) and 96 hours (median 4.67 versus 3.56, *p* = 0.03) [59], suggesting an inflammatory contribution to new-onset postprocedural AF. In another study with diabetic patients, NLR was significantly higher in those with AF than in the AF-free group (mean 2.87 ± 1.3 versus 2.2 ± 1.56, *p* = 0.019) and was an independent risk factor for AF (OR 3.486, *p* = 0.004) using 2.38 as cut-off [60].

### 3.2. Severity and Incidence of Stroke

A high NLR not only predicts higher incidence of AF but also is a predictor of disease severity and risk of stroke [61, 62]. In a study with 309 patients with nonvalvular AF, a high NLR (>2.59) was an independent risk factor for the presence of left atrial thrombus on transesophageal echocardiography (TEE) (OR 1.59; *p* < 0.02) [61]. In another study with TEE, a high NLR (>2.92) was a predictor of reduced (<10 cm/sec) left atrial appendage wall velocity (LAAWV) in patients with paroxysmal AF [63]. A large retrospective cohort including 32.912 patients with AF showed that each increase in NLR quartile above the lowest was associated with a significant increase in risk of stroke with HRs of 1.11 (0.91–1.35), 1.25 (1.03–1.51), and 1.56 (1.29–1.88) for the second, third, and highest quartiles, respectively; and adding NLR to CHA2DS2-VASc risk score improved the accuracy for prediction of stroke [62]. Even in those in oral anticoagulation, a high NLR level was a predictor of stroke [20].

### 3.3. Treatment Response and Mortality

A high NLR is also a predictor of poor response to treatment as shown in a study, where it predicted AF recurrence after successful cardioversion with amiodarone [64]. In another study with 251 patients with symptomatic AF who underwent cryoablation, a high preablation NLR (>3.15) was a predictor of postprocedural disease recurrence (HR 2.15, 95% CI 1.70 to 2.73, *p* < 0.001) [65]. Regarding mortality, no specific study relating NLR and increased mortality in patients with AF was found. However, a high NLR was an independent predictor of short- and long-term mortality in patients with stroke in general (irrespective of being cardioembolic or atherosclerotic) [24, 66, 67].  Table 2 summarizes the clinical studies that have assessed the role of NLR as a prognostic biomarker in AF.

## 4. Underlying Mechanisms, Pathways, and Relationship between Biomarkers in AF

In relation to the underlying mechanisms, despite the consistency of the studies regarding the epidemiological association between inflammation and AF, there is still a substantial scarcity of data in basic sciences giving the pathophysiologic background to this link. In the case of the CRP, an acute phase protein, it seems to be more a marker of underlying immune responses than an active participant in the pathogenesis of the disease. This is reinforced by the fact that genetic polymorphisms that are associated with the double increase in CRP showed no significant association with the AF [27]. On the other hand, multiple factors interfere with CRP that would be very difficult to control in primary studies, to evaluate possible pathophysiological nexus. For this reason, we focus our description more on those related to NLR, and IL-6, which are more than simple reflectors, seeming to be actively involved in the pathogenesis of AF.

Neutrophil-lymphocyte ratio is a derived marker, expressing an imbalance in leukocytes with the dominance of neutrophils over lymphocytes, which may be only the “tip of the iceberg” of a deeper imbalance in the immunologic response. This seems particularly true from the observation that a high NLR is associated with the excessive activation of interleukin-17 (IL-17) axis in AF [26], which is a cytokine produced mainly by T-helper 17 (Th17) a subset of T-helper cells. In fact, the differentiation of Th17 cells from naïve T cells is mediated largely by IL-6 [73–75], a cytokine produced mainly by macrophages, which are neutrophils infiltrating tissues. So, IL-6 would induce, at T-helper cells level, the polarization of the differentiation favoring the effectors Th17 cells over the regulatory T (Treg) cells [76]. Th17 cells produce IL-17 that, among other functions, is responsible for the increase in fibrosis, which is a crucial component in AF [77–80]. It is interesting that those diseases that have the IL-17 as a cornerstone of its pathophysiology, like psoriasis, or with significant increase of its levels such as nonalcoholic fatty liver disease and metabolic syndrome are associated with increased incidence of AF [17, 19, 81–83], suggesting a role of this cytokine as a mediator between AF and these clinical conditions.

The IL-17 is also associated with the upregulation of transforming growth factor beta (TGF-*β*) signaling pathways [84], another potent promoter of atrial fibrosis and consequent AF [85–87]. In addition, IL-17 stimulates the production of more proinflammatory cytokines such as tumor necrosis factor- (TNF-) *α* and IL-6 [88, 89] and regulates tissue infiltration by neutrophils and myocyte apoptosis, which can start and engage various other pathophysiological pathways including oxidative stress and hypercoagulability [90–92]. IL-17 induces the production of IL-6, a potent inductor of IL-17 synthesis, occurring in this way, a refeeding on the axis [73, 74].

The IL-6, as we described, plays a critical role in the regulation of IL-17 axis [73, 75]. IL-6, together with IL-23, induces the differentiation of Th17 cells from naïve T cells, at the same time that inhibits TGF-beta-induced Treg differentiation, favoring, in this way, the T17/Treg imbalance [73, 74, 76]. There is still a significant gap between the epidemiological evidence of inflammation in AF and the current understanding of underlying physiopathological basis. The clear understanding of this association is still an object of future studies from basic science to clinical practice level.

## 5. Concerns and Limitations of the Use of Inflammatory Biomarkers in AF

The main concerns and doubts that arises from the potential use of inflammatory biomarkers in AF is about the additional value of using a panel of two or more biomarkers in predicting AF outcomes than the isolated use, and if there is a superiority of a biomarker in relation to others in AF. No primary study has evaluated the additional value of two or more biomarkers in comparison with the isolated use. Even studies that studied the correlation between biomarkers [11, 14, 38] did not evaluate the additive effect of them to predict outcomes. Despite this gap, it is very likely that in clinical practice a panel of 2 biomarkers or more may be better than the isolated use of one, for predicting AF-related outcomes. So, the evaluation of the additive effect of combined use should be a subject for future studies. On the other hand, the primary studies are very controversial about the superiority of a biomarker in relation to others. So, in light of the current literature, there are no sufficient data to support such point, highlighting only that NLR is more easily accessible and inexpensive than other biomarkers as hs-CRP and IL-6.

## 6. Conclusion and Future Directions

The review of the available evidence shows that inflammatory biomarkers such as IL-6, IL-17, and NLR have a crucial role in the pathogenesis of AF. They represent an additional, noninvasive tool, with good performance, to predict new-onset disease, persistence, treatment response, recurrence, the risk of complications, and mortality in AF. The available evidence suggests that NLR, a simpler, widely available, and inexpensive biomarker, is a predictor of incidence, treatment success, recurrence, and thromboembolic complications.

Next studies should be addressed to clarify the underlying mechanisms in AF, to establish the additional value of using a panel of two or more biomarkers in predicting AF outcomes, to evaluate the superiority of a biomarker in relation to others, and to test the value of different biomarkers in different situations in the setting of AF.

## Figures and Tables

**Table 1:** Clinical studies on the predictive value of inflammatory biomarkers (other than NLR) in atrial fibrillation (chronological order).

Study (year) [ref]	Biomarker(s)	Number of patients	Threshold	Assessment period	Results
Conway et al. (2004) [11]	IL-6 and CRP	106 patients with chronic AF and 41 healthy controls	Median comparison between groups	At baseline of the study	Patients with AF had significantly higher levels of IL-6 (median 24 versus 3 pg/mL, *p* = 0.034) and CRP (median 0.27 versus 0.13 mg/dL, *p* = 0.003), compared with controls. Plasma IL-6 levels were higher among AF patients at “high” risk of stroke using risk scores (*p* = 0.003)

Thambidorai et al. (2004) [31]	hs-CRP	104 patients with AF who underwent TEE	Median comparison between groups	CRP measured ≤1 week after TEE	Patients with identified thromboembolic risk factors on TEE had greater CRP levels than those without (1.00 versus 0.302 mg/dL). CRP also correlated with clinical stroke risk factors

Psychari et al. (2005) [39]	CRP and IL-6	90 patients with AF (70 with persistent AF who underwent PCV and 20 with permanent AF) and 46 controls	Mean comparison between groups	6 hours after CV or in the morning hours after fasting	Compared with controls patients with AF had increased CRP (mean 5.7 versus 2.3 mg/L, *p* = 0.002) and IL-6 (mean 8.3 versus 2.9 pg/mL, *p* < 0.001). There was positive relation between LAD and inflammatory markers (CRP [*R* = 0.37, *p* < 0.001] and IL-6 [*R* = 0.46, *p* < 0.001])

Malouf et al. (2005) [33]	hs-CRP	67 patients with AF or atrial flutter who underwent successful ECV	Mean comparison between groups	Before ECV	Pre-CV hs-CRP levels were an independent predictor of arrhythmia recurrence (OR 2.19, 95% CI 1.05–4.55, *p* = 0.036) even after adjusting for confounders

Watanabe et al. (2006) [32]	hs-CRP	106 patients with AF who underwent ECV	≤0.12 mg/dL for CV success and ≥0.06 mg/dL for recurrence	Immediately prior to ECV	A lower hs-CRP (≤0.12 mg/dL) was an independent predictor of successful ECV (OR 0.33, 95% CI 0.21–0.51). In turn, a high hs-CRP was the only independent predictor of AF recurrence (OR 5.30, 95% CI 2.46–11.5) using a cut-off value of hs-CRP ≥ 0.06 mg/dL, and after adjustment for coexisting cardiovascular risks

Lip et al. (2007) [40]	CRP and CD40	880 subjects with AF from SPAF III clinical trial	Multiple cut-offs (tertiles)	Within 30 days of enrollment or after 3 months in the study	Patients with moderate to high stroke risk (measured by CHADS2 score and NICE criteria) had the highest levels of CRP (Kruskal Wallis test, *p* < 0.001). All-cause mortality (log rank test, *p* = 0.001) and vascular events (*p* = 0.05), but not stroke, were more common in patients with high CRP levels during a mean follow-up of 453 (±229) days. Soluble CD40 ligand was not related to prognosis

Liu et al. (2007) [41]	CRP	A meta-analysis of 7 studies with 420 AF patients who underwent successful ECV	Mean difference between groups	At baseline of primary studies	Atrial fibrillation relapsed in 229 patients. Baseline CRP levels were greater in patients with AF recurrence than in those without (SMD 0.35 units, 95% CI 0.01–0.69)

Fujiki et al. (2007) [38]	IL-6 and CRP	35 patients with AF who underwent successful PCV	Mean comparison between groups	After pharmacological restoration of SR	During the 1-year follow-up period, 15 patients presented recurrence of AF. Patients with AF recurrence had significantly higher plasma levels of both IL-6 (mean 1.84 ± 0.66 versus 1.19 ± 0.51 ng/L, *p* < 0.005) and CRP (1.24 ± 0.79 mg/L versus 0.59 ± 0.40, *p* < 0.005) than those without. There was a significant positive correlation between levels of IL-6 and CRP

Henningsen et al. (2009) [42]	IL-6 and hs-CRP	56 patients with persistent AF who underwent successful ECV	2.8 pg/mL for IL-6 and 3.0 mg/L for hs-CRP (analysis included median comparison)	Before CV and after 1, 30, and 180 days	After 180 days of follow-up, the recurrence rate was 68%. Patients with recurrence of AF had significantly higher hs-CRP (2.0 versus 1.25 mg/L, *p* < 0.001) and IL-6 (2.75 versus 1.96 pg/mL, *p* < 0.001) than those who maintained SR. Baseline IL-6 was the only independent predictor of recurrent AF (*p* = 0.04) in a multivariate Cox analysis

Henningsen et al. (2009) [43]	IL-6 and hs-CRP	46 patients with paroxysmal or persistent AF who underwent RFCA	Median comparison between groups	Before the first ablation procedure and at follow-up visits	After 12 months of follow-up, the recurrence rate was 59%. Patients with recurrence of AF had significantly higher IL-6 (1.4 versus 0.9 pg/mL, *p* = 0.007) and hs-CRP (2.2 versus 0.7 mg/L, *p* = 0.018) at baseline than those who maintained SR. IL-6 concentration prior to ablation was an independent predictor of recurrent AF (*p* = 0.027)

Lin et al. (2010) [15]	hs-CRP	137 patients with AF who underwent mapping and catheter ablation	2.92 mg/L	Before the first ablation procedure	Higher hs-CRP was associated with an increased frequency of nonpulmonary vein ectopies (34.4% versus 17%, *p* = 0.034) and was an independent predictor of recurrence (*p* = 0.021) in a multivariable regression model after adjusting for other potential covariates

Cianfrocca et al. (2010) [44]	CRP	150 patients with persistent nonvalvular AF, who underwent TEE prior to CV	3 mg/L (analysis included mean comparison between groups)	Before CV	C-reactive protein was significantly associated with thrombus and/or dense SEC (OR 3.41, 95% CI 1.2–9.8)

Maehama et al. (2010) [45]	CRP	A total of 165 patients with nonrheumatic AF	Median comparison between groups	Within 1 week before TEE	Patients in the high-risk group according to CHADS2 score had significantly greater CRP levels than those in the intermediate- and low-risk groups (0.80 mg/dL versus 0.16 mg/dL versus 0.08 mg/dL, *p* < 0.01, resp.). And the incidence of LA SEC and LA thrombus on TEE increased with an increasing CHADS2 score

Marott et al. (2010) [27]	CRP	46,876 individuals from 2 large studies (including 2,111 with AF)	Multiple cut-offs (quintiles)	NA	The highest CRP quintile was associated with increased risk of atrial fibrillation compared with the lower quintile (OR 2.19, 95% CI 1.54–3.10). However, CRP did not fulfill the causality criterion, whereas its elevation by genetically CRP did not increase atrial fibrillation risk

You et al. (2010) [14]	CRP, IL-6, and Cystatin C	103 AF patients (28 with AF complicated by ischemic stroke) and 112 controls	Median comparison between groups	At baseline	AF patients had higher levels of hs-CRP (*p* = 0.004), IL-6 (*p* = 0.000), and cystatin C (*p* = 0.000) than control subjects. Plasma hs-CRP level was also higher in patients with AF complicated by ischemic stroke compared with those with simple AF (*p* = 0.036)

Luan et al. (2010) [36]	IL-18 and MMP-9	56 patients with AF and 26 controls	Mean or median comparison between groups	At first 24 hours after admission	IL-18 was significantly higher in patients with AF than in controls (471.50 ± 144.91 versus 232.20 ± 55.33 pg/mL; *p* < 0.0001). MMP-9 (OR = 1.02, 95% CI: 1.00–1.03, *p* = 0.012) and IL-18 were independently associated with AF (OR = 1.02, 95% CI: 1.01–1.03, *p* = 0.001). Interleukin-18 levels were also higher in persistent AF patients than in those with paroxysmal AF (*p* = 0.0011)

Celebi et al. (2011) [46]	hs-CRP	216 patients with persistent AF who underwent CV	1.85 mg/dL (the analysis included mean comparison between groups)	Prior to and 1, 2, 7, and 30 days after CV	The basal hs-CRP levels were higher in patients with an AF relapse than in those without (1.68 ± 0.57 versus 1.12 ± 0.53 mg/dL; *p* < 0.01). By multivariate Cox analysis, the independent predictors of AF relapse time points were the basal and day-2 hs-CRP levels

Liu et al. (2011) [47]	hs-CRP	121 patients with AF (paroxysmal/persistent AF: 77/44) who underwent CPVI	1.41 mg/L (the analysis included median comparison between groups)	On the morning of admission, before the procedure	The plasma hs-CRP concentration was significantly higher in the group with AF recurrence than in the nonrecurrent one (median 2.22 mg/L versus 0.89 mg/L, *p* < 0.001). A higher hsCRP was a significant predictor of AF recurrence in overall (OR 5.10, 95% CI 2.14–12.11, *p* < 0.001) and in subgroups of paroxysmal (OR 4.12, 95% CI 1.36–12.47, *p* = 0.012) and persistent AF (OR 16.37, 95% CI 2.52–56.42, *p* = 0.003)

Kim et al. (2011) [48]	TGF-*β* and TIMP-1	242 patients with AF (155 paroxysmal AF, 87 persistent AF) who underwent CA	10.0 ng/mL for TGF-*β* and 1.1 ng/mL for TIMP-1	Biomarker measurement, LA voltage map, and 3D-CT before CA	Patients with higher TGF-*β* (≥10.0 ng/mL) had lower mean LA voltage (*p* = 0.014) and greater LA volume (*p* = 0.022) than those with lower levels. Similarly, patients with higher TIMP-1 (≥1.1 ng/mL) had lower mean LA voltage (*p* = 0.019) than those with lower levels. This reflects that higher plasma concentrations of this markers are closely related with LA electroanatomical (voltage and structural) remodeling

Kinoshita et al. (2011) [49]	CRP	552 patients who underwent coronary bypass surgery, analyzed retrospectively	Multiple cut-offs (the analysis included median comparison between groups)	Preoperative	AF occurred in 21.9% of patients after surgery. The median value of CRP was higher in patients who developed AF than in those who did not (2.2 versus 1.3, *p* = 0.001). This association persisted after adjustment for confounders (HR 1.43, 95% CI 1.22–1.97 per 1 SD increase in CRP, and HR 2.88, 95% CI 1.67–4.97 for CRP within 3.0–10.0 versus <1.0 mg/dL)

Hermida et al. (2012) [50]	hs-CRP	293 with a history of AF	Multiple cut-offs (tertiles)	At visit 4	During a median follow-up of 9.4 years, hs-CRP was associated with increased risk for all-cause mortality comparing the highest versus the lowest tertiles (HR 2.52, 95% CI 1.49–4.25, *p* < 0.0001) after adjusting for potential confounders

Peña et al. (2012) [29]	hs-CRP	17,120 participants without prior history of arrhythmia	Multiple cut-offs (<3.2, 3.2–5.8, and ≥5.8 mg/L)	At study baseline	Each increase in hs-CRP tertile from the lowest was associated with a 36% increase in the risk of developing AF (HR 1.37, 95% CI: 1.16–1.60, *p* < 0.01), with an HR of 1.96 (95% CI: 1.40–2.75, *p* < 0.01) when comparing the highest hs-CRP tertile with the lowest

Roldán et al. (2012) [37]	IL-6	930 patients with permanent/paroxysmal AF in chronic anticoagulation	Multiples (3.35 pg mL^−1^ for CVE, 4.16 pg mL^−1^ for mortality)	At baseline	During a median follow-up of 957 (784–1087) days, 107 adverse cardiovascular events occurred (3.14%/year), which included 37 stroke/TIA events (1.5%/year). On multivariate analysis, a high IL-6 was associated with adverse cardiovascular events (OR 1.97, 95% CI 1.29–3.02, *p* = 0.002) and all-cause mortality (HR 2.48, 9% CI 1.60–3.85, *p* < 0.001)

Barassi et al. (2012) [51]	hs-CRP	57 patients with AF who underwent ECV	2.99	Before and 3 weeks after ECV	CRP levels (>2.99–3.10 mg/L) were significantly associated with AF recurrences (OR, 14.93, 95% CI 3.90–57.19, *p* < 0.001)

Mazza et al. (2013) [52]	hs-CRP	92 patients with AF and hypertension who underwent ECV	0.30 * *mg/dL	Before CV	A higher hs-CRP (>0.30 mg/dL) was associated with *p*-wave alterations such as *P* maximum above 120 ms (*p* = 0.002) and *P* dispersion above 40 ms (*p* = 0.0006) that are useful predictors of AF recurrence

Parashar et al. (2013) [53]	hs-CRP and NT-proBNP	2,370 patients with AMI, but without AF from TRIUMPH study	Median comparison between groups	At study baseline	There was a 15% increase in the rate of AF (OR 1.15, 95% CI 1.02–1.30, *p* = 0.02), for each 2-fold increase in CRP. Similarly, for every 2-fold increase in NT-proBNP, there was an 18% increase in the rate of AF (OR 1.18, 95% CI 1.03–1.3, *p* < 0.02)

Sinner et al. (2014) [54]	CRP and BNP	18,556 Whites and African Americans from three primary studies (ARIC, CHS, and FHS)	Multiple cut-offs (each 1-SD increase)	At the index visit	1,186 new cases occurred in five years of follow-up. CRP was significantly associated with AF incidence (HR 1.18, 95% CI 1.11–1.25, *p* < 0.0001), per 1-SD increase of ln-transformed values, as was BNP (HR 1.66, 95% CI 1.56–1.76, *p* < 0.0001)

Dewland et al. (2015) [55]	CRP IL-6, TNF-*α*, TNF-*α* SR I, and others	2,768 participants without AF (43% Black) from Health ABC Study	Multiple cut-offs (depending on the biomarker)	At the baseline study visit	During a median follow-up of 10.9 years, 721 developed incident AF. Adiponectin, CRP, IL-6, TNF-*α*, TNF-*α* SR I, and TNF-*α* SR II concentrations were each higher among Whites and independently associated with a greater risk of incident AF. Together, these inflammatory cytokines mediated 42% (95% CI 15 to 119%, *p* = 0.004) of the adjusted white race associated AF

Aulin et al. (2015) [56]	IL-6 and hs-CRP	6,187 patients with nonvalvular AF from the RE-LY study	Multiple cut-offs (quartiles)	Before start of study intervention	In patients with AF, IL-6 was independently associated with stroke or systemic embolism (*p* = 0.0041), major bleeding (*p* = 0.0001), vascular death (*p* < 0.0001), and a composite thromboembolic outcome (ischemic stroke, systemic embolism, myocardial infarction, pulmonary embolism, and vascular death) (*p* < 0.0001), after adjusting for clinical risk factors. Similarly, CRP was independently related to myocardial infarction (*p* = 0.0047), vascular death (*p* = 0.0004), and the composite thromboembolic outcome (*p* = 0.0001)

Amdur et al. (2016) [35]	IL-6	3,762 adults with CKD enrolled in the CRIC study	Multiple cut-offs (tertiles)	At baseline	Plasma IL-6 level was significantly associated with presence of AF at baseline (OR 1.61, 95% CI 1.21– 2.14, *p* = 0.001) and was an independent predictor of NOAF (OR 1.25, 95% CI, 1.02–1.53, *p* = 0.03) during a mean follow-up of 3.7 years, after adjusting for confounders

Negreva et al. (2016) [57]	hs-CRP	51 patients with AF and 52 controls	Mean comparison between groups	At hospital admission, 24 hours, and 28 days after SR restoration	hs-CRP concentrations were higher in patients with AF than in controls at baseline (mean 8.12 ± 0.82 versus 5.57 ± 0.21 mg/L, *p* = 0.003), and the difference persisted 24 hours after SR restoration (8.16 ± 0.71 versus 5.57 ± 0.21 mg/L, *p* < 0.001)

Hijazi et al. (2016) [58]	IL-6 and CRP	14,954 patients with AF on anticoagulation from the ARISTOTLE trial	Multiple cut-offs (quartiles)	At randomisation	There was a significant association between IL-6 and CRP and all-cause mortality independent of clinical risk factors and other biomarkers (HR 1.93, 95% CI 1.57–2.37 for IL-6, and HR 1.49 95% CI 1.24–1.79 for CRP, comparing the highest with the lowest quartiles). However, there were no associations with the risk of stroke or major bleeding

3D-CT: three-dimensional computed tomography; AF: atrial fibrillation; AMI: Acute Myocardial Infarction; ARIC: Atherosclerosis Risk in Communities Study; ARISTOTLE: Apixaban for Reduction in Stroke and Other Thromboembolic Events in Atrial Fibrillation; CA: catheter ablation; CABG: coronary artery bypass grafting; CAD: coronary artery disease; CHADS2: one point for congestive heart failure, hypertension, age > 75, diabetes mellitus, and two points for prior stroke or transient ischemic attack; CHS: Cardiovascular Health Study; CI: confidence interval; CKD: chronic kidney disease; CPVI: circumferential pulmonary vein isolation; CV: cardioversion; CVE: cardiovascular events; ECV: electrical cardioversion; FHS: Framingham Heart Study; Health ABC: Health, Aging, and Body Composition; HR: hazard ratio; IL-17A: interleukin-17A; IL-6: interleukin-6; LAAWV: left atrial appendage wall velocity; LAD: left atrial diameter; MACE: major adverse cardiovascular events; MMP-9: matrix metalloproteinase-9; NA: not available; NICE: National Institute for Health and Clinical Excellence; NOAF: new-onset atrial fibrillation; NT-proBNP: NT-pro-brain natriuretic peptide; OR: Odds Ratio; PCI: percutaneous coronary intervention; PCV: pharmacological cardioversion; POAF: postoperative atrial fibrillation; RE-LY study: “Randomized Evaluation of Long-term anticoagulant therapY” study; RFCA: radiofrequency catheter ablation; SAFHIRE: Study of Atrial Fibrillation in High-Risk Elderly; SEC: spontaneous echo contrast; SMD: standardized mean difference; SPAF: Stroke Prevention in Atrial Fibrillation; sPsel: soluble P-selectin; SR: sinus rhythm; STEMI: ST-segment elevation myocardial infarction; TEE: transesophageal echocardiography; TGF-*β*: transforming growth factor-*β*; TIMP-1: tissue inhibitor of metalloproteinase-1; TNF-*α*: tumor necrosis factor alpha; TNF-*α* SR I: tumor necrosis factor alpha soluble receptor I; TNF-*α* SR II: tumor necrosis factor alpha soluble receptor II; vWf: von Willebrand factor.

**Table 2:** Clinical studies on the predictive value of the neutrophil-lymphocyte ratio as a biomarker in atrial fibrillation.

Study [ref]	Year	Number of patients	Threshold	Assessment period	Results
Gibson et al. [21]	2010	275 patients without previous atrial arrhythmia, undergoing CABG	Median comparison between groups	Preoperatively and on postoperative day 2	The incidence of AF was greater in groups with higher preoperative NLR (median 3.0 versus 2.4, *p* = 0.001) and postoperative NLR (median 9.2 versus 7.2, *p* < 0.001)

Ertaş et al. [20]	2013	126 patients with nonvalvular AF	Mean comparison among subjects with or without stroke	At admission	In patients with nonvalvular AF, mean NLR was significantly higher among subjects with stroke compared to individuals without a stroke (5.6 versus 3.1)

Canpolat et al. [65]	2013	251 patients with symptomatic AF who underwent cryoablation	3.15	Preprocedural	Patients with a high preablation NLR (>3.15) had a 2.5-fold increased risk of AF recurrence after successful cryoablation

Im et al. [68]	2013	499 patients who underwent RFCA for paroxysmal or persistent AF	5.6	At baseline and on day 1 after RFCA	In multivariate analysis, a high post-NLR was an independent predictor for early recurrence after RFCA (HR 1.09; *p* 0.047). Patients with higher NLR (>5.6) had significantly lower AF-free survival on Kaplan-Meier (K-M) curve

Sahin et al. [60]	2013	144 diabetic patients (72 with and 72 without AF)	2.38 (analysis included mean comparison between groups)	Retrospectively recorded from patient files	The mean NLR was significantly higher in diabetic patients with AF than in those without (mean 2.87 versus 2.2, *p* = 0.019). Using a cut-off point of 2.38 NLR was associated with AF (OR 3.486, *p* = 0.004)

Trivedi et al. [70]	2013	165 patients with paroxysmal AF, who underwent RFCA	3.08 (analysis included mean comparison between groups)	One day prior to ablation	Baseline NLR was high in patients with AF recurrence (mean 3.2 versus 2.5, *p* < 0.001). A high baseline NLR (>3.08) was a significant predictor of postablation AF recurrence (HR 1.99, 95% CI 1.33–2.96, *p* = 0.001)

Guo et al. [69]	2014	379 lone AF patients who underwent catheter ablation	5.15 (analysis included mean comparison between groups)	Before and after catheter ablation	The patients who developed AF recurrence had a higher postablation NLR than patients with no recurrence (5.74 versus 4.66, *p* < 0.001). A high postablation NLR (>5.15) was an independent predictor of AF recurrence (HR 1.514, 95% CI 1.36–1.68, *p* < 0.001)

Acet et al. [71]	2014	A total of 197 subjects (71 with paroxysmal, 63 with persistent/permanent AF, and 63 AF-free controls)	2.1 (analysis included mean comparison between groups)	At baseline	Higher NLR (>2.1) had a significant relationship with nonvalvular AF (OR 11.31, *p* < 0.001) compared with control group; and the mean value was significantly higher in those with persistent/permanent compared to those with paroxysmal AF (3.4 ± 0.6, versus 2.5 ± 0.6, *p* < 0.001)

Nikoo et al. [26]	2014	112 AF patients and 107 controls	Mean comparison between groups	At baseline	A significant positive correlation was observed between NLR and increased interleukin-17 (IL-17A) in AF (*p* = 0.006). Elevated IL-17A, on the other hand, was significantly increased in patients with AF compared to controls (1.28 ± 3.5 versus 0.19 ± 0.64 pg/mL, *p* = 0.001)

Karavelioğlu et al. [64]	2015	218 patients restored to sinus rhythm with amiodarone	Mean comparison between groups	At admission	A high NLR was an independent predictor of AF recurrence (OR 1.584 [1.197–2.095], *p* = 0.001) after successful cardioversion with amiodarone

Yalcin et al. [61]	2015	309 patients with nonvalvular AF who underwent TEE	2.59	Before TEE	A high NLR (>2.59) was an independent risk factor for the presence of left atrial thrombus on TEE (OR 1.59; *p* < 0.02) in patients with nonvalvular AF

Saliba et al. [62]	2015	32.912 patients with AF	Multiple cut-offs in quartiles	Median NLR value of the tests performed in the year prior to study entry	Each increase in NLR quartile above the lowest was associated with a significant increase in the risk of stroke with HRs (95% CI) 1.11 (0.91–1.35), 1.25 (1.03–1.51), and 1.56 (1.29–1.88) for the second, third, and highest quartiles, respectively

Chavarria et al. [59]	2015	290 patients who underwent PCI for acute STEMI	Median comparison between groups	<6 hours preprocedural, <12, 48, and 96 hours postprocedural	Patients who developed AF (*n* = 40, 13.8%) had higher postcatheterization NLR at 48 hours (median 5.23 versus 3.00, *p* = 0.05) and 96 hours (median 4.67 versus 3.56, *p* = 0.03)

Fukuda et al. [63]	2015	120 patients with paroxysmal AF	2.92	At baseline	A higher NLR (>2.92) was a predictor of reduced LAAWV in patients with paroxysmal AF

Wagdy et al. [72]	2016	200 patients with STEMI	4.6	At admission	A higher NLR (>4.6) was an independent predictor of NOAF, no-reflow, and in-hospital MACE (OR 3.5, *p* = 0.02) in patients with STEMI, after adjustment for confounding factors

AF: atrial fibrillation; NLR: neutrophil-lymphocyte ratio; OR: Odds Ratio; TEE: transesophageal echocardiography; CABG: coronary artery bypass grafting; RFCA: radiofrequency catheter ablation; NOAF: new-onset atrial fibrillation; LAAWV: left atrial appendage wall velocity; NA: not available; STEMI: ST-segment elevation myocardial infarction; CI: confidence interval; IL-17A: interleukin-17A; MACE: major adverse cardiovascular events; PCI: percutaneous coronary intervention.
